# Mechanical Properties and Residual Stress Measurements of Grade IV Titanium and Ti-6Al-4V and Ti-13Nb-13Zr Titanium Alloys after Laser Treatment

**DOI:** 10.3390/ma14216316

**Published:** 2021-10-22

**Authors:** Magdalena Jażdżewska, Dominika Beata Kwidzińska, Wiktor Seyda, Dariusz Fydrych, Andrzej Zieliński

**Affiliations:** 1 Institute of Manufacturing and Materials Technology, Faculty of Mechanical Engineering and Ship Technology, Gdańsk University of Technology, 80-233 Gdańsk, Poland; dominika.trochowska@pg.edu.pl (D.B.K.); dariusz.fydrych@pg.edu.pl (D.F.); andrzej.zielinski@pg.edu.pl (A.Z.); 2 Office of Technical Inspection, 80-233 Gdańsk, Poland; wiktor.seyda@udt.gov.pl

**Keywords:** titanium, Ti-6Al-4V, Ti-13Nb-13Zr, laser treatment, surface engineering

## Abstract

Nowadays, surface engineering focuses on research into materials for medical applications. Titanium and its alloys are prominent, especially Ti-6Al-4V and Ti-13Nb-13Zr. Samples made of pure grade IV titanium and the titanium alloys Ti-6Al-4V and Ti-13Nb-13Zr were modified via laser treatment with laser beam frequency f = 25 Hz and laser beam power P = 1000 W during a laser pulse with duration t = 1 ms. Subsequently, to analyze the properties of the obtained surface layers, the following tests were performed: scanning electron microscopy, chemical and phase composition analysis, wetting angle tests and roughness tests. The assessment of the impact of the laser modification on the internal stresses of the investigated materials was carried out by comparing the values of the stresses of the laser-modified samples to those of the reference samples. The obtained results showed increased values of tensile stresses after laser modification: the highest value was found for the Ti-6Al-4V alloy at 6.7434 GPa and the lowest for pure grade IV titanium at 3.742 GPa. After laser and heat treatment, a reduction in the stress was observed, together with a significant increase in the hardness of the tested materials, with the highest value for Ti-6Al-4V alloy at 27.723 GPa. This can provide better abrasion resistance and lower long-term toxicity, both of which are desirable when using Ti-6Al-4V and Ti-13Nb-13Zr alloys for implant materials.

## 1. Introduction

In recent years, titanium has gained much interest in the technology world for its unique properties. In the earth’s crust it occurs in the form of minerals such as titanite, anatase, ilmenite, brookite, perovskite and rutile, with a content of only 0.61% (the ninth most abundant element) [1]. The pure metal and its alloys have a wide range of applications in many areas, inter alia: biomedical, chemical, offshore, transportation (aviation and automotive), energy and recreation [2,3,4,5]. Titanium is completely resistant to moist hydrochloric acid, nitric acid, chlorine compounds, sulfides, sulfur, ammonia and hydrogen peroxide. Therefore, it is used in the construction of pumps intended for acids. Since titanium shows weak magnetic properties, it is a good material for submarines, offering a higher resistance to magnetic mines. Additionally, patients with titanium-containing implants can undergo magnetic resonance imaging. Titanium and its alloys are also used in the production of sports equipment, such as bicycle frames, tennis rackets, competitive sleds, golf clubs, etc. [6,7,8,9].

Titanium is characterized by the highest biotolerance, high biocompatibility and the lowest Young’s modulus among currently used metallic biomaterials, and it also shows corrosion resistance in the aggressive environment of the human body [10,11,12]. As a result, it is successfully used in facial plates, pacemakers, endoprostheses and dental implants [13,14]. 

One of the most commonly used titanium alloys is the Ti-6Al-4V alloy. It is a two-phase alloy, characterized by a low density and very good corrosion resistance. It also shows good machinability and good mechanical properties, especially high mechanical strength in relation to its density [15,16,17]. However, recent research proves that the main elements contained in the Ti-6Al-4V alloy also have negative impacts on the human body. Aluminum may cause increased osteoporotic phenomena or even damaged nerve cells. Furthermore, it affects the deterioration of the activity and function of enzymes and neurotransmitters, and this may initiate Alzheimer’s disease. The second main element, vanadium, may respond to unwanted cytological reactions and induce neurogenic disorders [2]. The Ti-6Al-4V alloy is an excellent material for applications requiring direct contact with tissue or bone. Unfortunately, it has low shear strength, and this is undesirable for bone plates or screws. To increase the hardness or abrasion resistance and the biological activity of the Ti-6Al-4V alloy, surface modifications are carried out. Thermal–chemical treatment (nitriding and oxy-nitriding processes) plays an increasingly important role in surface engineering. Such modification allows for high abrasion resistance and good biocompatibility, which is especially important for medical applications, i.e., long-term implants [18,19].

The Ti-13Nb-13Zr alloy is an innovative material with zero toxicity to human tissues, unlike Ti-6Al-4V which contains aluminum and vanadium [20,21,22,23]. The advantages of this alloy include good mechanical properties, with relatively low density, excellent biocompatibility and good abrasion resistance. Both the Ti-6Al-4V and Ti-13Nb-13Zr alloys have a modulus of elasticity similar to that of human bone. Additional modifications of the surface of titanium alloys enable appropriate osseointegration [24,25].

The surface of the titanium alloys can be modified by laser treatment [12,15,18,26,27,28]. This type of modification is characterized by no contact and remote control of the laser beam path, thus maintaining the high cleanliness of the treated surface. In addition, the laser beam can be focused to a very small size, so that a very high power density and selective beam influence on a precisely defined area of the surface of the material can be used. The advantage of the process is that it provides a large amount of energy in the very short time of the laser pulse. The characteristics of the laser treatment include the minimization of chemical pollution and the leveling of oxidation processes [29,30,31,32].

In the process of heat treatment, elasto-plastic deformations are induced in the treated surface. The size and extent of these deformations depend on the physical properties of the material and the heating and cooling temperatures. Stress is caused by the temperature difference and the change in microstructure between the surface and the core of the material [33].

Compressive residual stresses are important for increasing the fatigue strength of the material. By heating the surface, stresses tend to change from stretching to compression as the bead of the laser increases. On the other hand, as a result of cooling after laser treatment the remelted zone shrinks and tensile stresses form, which may cause the fracture of brittle materials [33]. Research has shown that laser treatment influences the appearance of compressive stresses up to a certain depth in the melted zone.

This research includes investigations of the effects of laser treatment on the microstructure of the remelted layer, the roughness, the wettability and the stress level within the surface layer of two titanium alloys: Ti-6Al-4V and Ti-13Nb-13Zr. The Ti-6Al-4V alloy is, together with medical titanium, the material most often used for load-bearing implants [34] and recently for making 3D-printed scaffolds [35,36] or for laser sintering or surface treatments [37,38]. On the other hand, the Ti-13Nb-13Zr alloy is now generally acknowledged as the safer alloy because it lacks the two harmful elements Al and V [35], and it has higher mechanical strength. However, although this alloy has been proposed for, e.g., dental implants [39] and bone implants [40], the laser surface modification of this alloy has not been discussed in the literature. The purpose of this study is therefore to present new results for the Ti-13Nb-13Zr alloy and to compare them to those for the more generally studied Ti-6A-l4V alloy, to consider whether the latter alloy may be substituted by the safer material when it is subjected to laser modification. 

The aim of the research was to analyze the influence of laser modification on selected properties of the obtained surface layers for samples made of titanium and the two alloys Ti-6Al-4V and Ti-13Nb-13Zr. The properties of the modified outer layers were compared, and their possible applications were determined.

## 2. Materials and Methods

### 2.1. Materials

Chemically pure grade IV titanium—base material BM1 (ChM, Białystok, Poland) and the titanium alloys Ti-6Al-4V—base material BM2 and Ti-13Nb-13Zr—base material BM3 were used. A rectangular sample of the Ti-6Al-4V alloy was cut from a 12 mm thick sheet, manufactured by TIMET, Birmingham, UK and delivered already annealed at 750 °C. The Ti-13Nb-13Zr (SeaBird Materials Co., Baoji, China) alloy sample was obtained by cutting a 30 mm diameter round bar with a wire saw. Then, it was divided into four 4 mm thick quadrants. The quarter circles were used in the research. Selected mechanical properties and the chemical compositions of the tested samples are presented in Table 1 and Table 2.

### 2.2. Preparation of Samples

For grinding all samples (before laser treatment) on a grinder and polisher (SAPHIR 330, ATM GmbH, Mammelzen, Germany), sandpapers with a grade of 500 and then 800 were used. In order to test the thickness of the melted layers, sample cross sections were made. Using a mounting press (OPAL 410, ATM ATM GmbH, Mammelzen, Germany), the samples were embedded in phenolic resin (Verte 602, Lamplan, Gaillard, France) for 20 min. Then, the attached samples were ground using sandpapers of grades 120, 240, 600 and 1200 and polished with sandpaper of grade 2000. The final stage of the preparation was etching the sample in order to accurately measure the thickness of the melted layers. Kroll’s reagent (0.06 mL of HF and 0.2 mL of HNO_3_ made up to 50 mL with distilled water) was used for etching. 

### 2.3. Laser Treatment

The laser modification was carried out using a Nd:YAG pulse laser (Trulaser Station 5004, TRUMPF, Ditzingen, Germany) which enabled the surface treatment of materials using a pulse mode. Samples made of technically pure grade IV titanium and Ti-6Al-4V and Ti-13Nb-13Zr alloys were subjected to laser modification using the process parameters presented in Table 3. To protect the surface against oxidation, an argon 5.0 (Linde Gaz Poland Ltd., Krakow, Poland) protective atmosphere was used.

**Figure 1:**
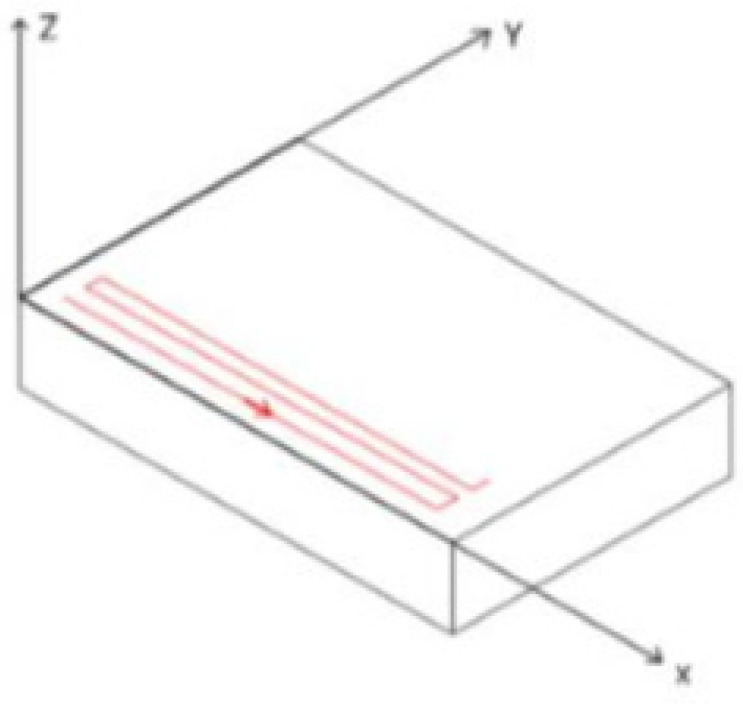
Diagram showing the path of the applied laser treatment.

The laser surface modification process was programmed using G-code. In one cycle, the laser beam first moved linearly along the *X*-axis of the sample, then stopped and moved a short distance along the *Y*-axis of the sample (up). Finally, the laser was moved linearly in a direction coinciding with the *X*-axis of the sample, continuing in this way until the end of the cycle. Figure 1 shows the path of the laser beam. The cycle was repeated 18 times. 

### 2.4. Analysis of Microstructure and the Chemical Composition of the Obtained Outer Layers with an SEM Microscope

A scanning electron microscope (SEM JEOL JSM-7800 F, JEOL Ltd., Tokyo, Japan) was used to analyze the microstructure. An X-ray energy dispersion spectrometer (EDS) (Octene Elite 25, EDAX, Mahwah, NJ, USA) is an SEM microscope attachment that uses backscattered electrons to determine the chemical composition.

### 2.5. The Phase Composition of the Obtained Outer Layers with XRD

The samples prepared from grade IV titanium and Ti-6Al-4V and Ti-13Nb-13Zr alloys were tested using an X-ray diffractometer (X’ Pert Pro, Philips, Almelo, The Netherlands) with a copper lamp. The X-ray diffraction was conducted using a monochromatic source, i.e., CuKα radiation (λ = 1.544 Å). The specimens were scanned from 20° to 90° at a scanning rate of 0.02°/s.

### 2.6. Roughness Studies

The roughness of the obtained layers was measured using a contact profilometer with EVOVIS software (1.38.02) (Hommel Etamic Waveline, Jenoptic, Jena, Germany). The system allowed automatic generation of the charts shown, i.e., base, raw and waviness profiles. During the analysis, the parameter Ra was determined.

### 2.7. Contact Angle Studies

A goniometer (contact angle goniometer, Zeiss, Oberkochen, Germany) was used to test the surface wettability using the falling drop method. Pictures of the water drop shape after t = 5 s were taken, recorded by the digital camera of the device. On the basis of these, it was possible to measure the right and left contact angles, from which the average contact angle for each of the tested surfaces was determined.

### 2.8. Analysis of Mechanical Properties

Nanoindentation tests were performed using the NanoTest^TM^ Vantage device (Micro Materials, Wrexham, Great Britain). A Berkovich diamond indenter with an apex angle of 124.4° was used for the analysis. The samples were tested before and after laser treatment. The parameters of the nanoindentation tests are shown in Table 4.

The modulus of elasticity was calculated after transforming the formula:

(1) $$\frac{1}{E_{r}}=\frac{1-v^{2}}{E}+\frac{1-v_{i}^{2}}{E_{i}}$$

where $v_{i}$ is the nanoindenter Poisson’s ratio (accepted as 0.07), v is the Poisson’s ratio of tested materials (accepted for each sample as 0.27), E is the elastic modulus of the tested samples, $E_{i}$ is the Young’s modulus of the nanoindenter (accepted as 1140 GPa) and $E_{r}$ is the reduced modulus of elasticity.

The final equation from which the reduced Young’s modulus was determined is:

(2) $$E=\frac{E_{i}\cdot E_{r}\cdot \left(1-v^{2}\right)}{E_{i}-E_{r}\cdot \left(1-v_{i}^{2}\right)}$$

### 2.9. Residual Stress Analysis

To assess the material residual stress, the nanoindentation method was used. All samples were measured before laser modification, after laser melting and after stress relief annealing. For each series of measurements, the mean value of the force for which a specific depth was reached on the material surface was determined. This was 300 nm in the case of samples where the maximum force value was equal to 50 mN, and 300 and 1000 nm when measured with a maximum force of 200 mN. Subsequently, the area of the Berkovich print at its recess in the material was estimated for each depth. The stress was defined as the quotient of the difference in the loads and the surface area, where the difference in the loads was measured before and after stress relief annealing for the grade IV titanium, Ti-6Al-4V and Ti-13Nb-13Zr samples and also for the same set of laser-melted materials. Formula 3 was used [41]:

(3) $$\sigma =\frac{\Delta P}{A}$$

where ΔP is the difference in the loads causing the same displacement of the indenter (for samples before and after stress relief annealing) and A is the surface area of the Berkovich indenter impression.

The common measurement procedure and Formulas (4)–(7) were used to determine the surface area of the print [41,42,43].

(4) $$h_{c}={\left(\frac{P_{max}}{3\sqrt{3}H\;tan^{2}\theta }\right)}^{\frac{1}{2}}$$

(5) $$E^{*}=\frac{dP}{dh}\cdot \frac{1}{2}\cdot \frac{\sqrt{\pi }}{\sqrt{A}}$$

(6) $$A=3\sqrt{3}h_{c}^{2}tan^{2}\theta =24.49h_{c}^{2}$$

(7) $$h_{c}=\frac{h_{t}+h_{r}}{2}$$

where $h_{t}$ is the maximum penetration depth of the indenter during the measurement and $h_{r}$ is the depth at which the indenter will remain when the force decreases to 0. 

In order to relax the internal stresses created in the surface layer after the laser modification, a heat treatment was carried out. When removing stresses, the most important parameters significantly influencing each other are the temperature and the annealing time. The same heat treatment effect can be obtained by increasing the temperature and reducing the annealing time, and vice versa. In the case of stress relief annealing, the process temperature should be in the range of 50–200 °C below the recrystallization temperature of the given material. On the other hand, the soaking time affects the degree of accuracy of relaxation and should not be too short. A long annealing time in a given temperature range does not adversely affect the strength and ductility of the material. In the case of titanium alloys, the cooling rate is not critical. However, the homogeneity is important. Therefore, it is recommended to cool these alloys evenly in air or in the furnace.

The melted and reference samples of each of the tested materials were annealed. The heat treatment was performed using a PTF 15/75/610 tube furnace from Protherm Furnaces, Ankara, Turkey, with a power of 7 kW. 

The annealing parameters were selected appropriately for each of the materials separately, based on literature data [44]. To prevent surface oxidation, all samples were annealed in vacuo and slow furnace cooling was applied. The precise heat treatment process parameters for each sample are shown in Table 5. A heating rate of 100 °C per h was used for all samples.

## 3. Results

### 3.1. Analysis of Macrostructure

**Figure 2:**
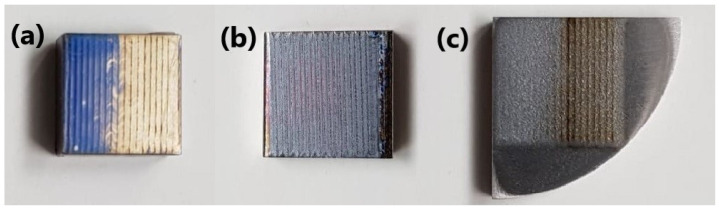
The samples after laser treatment: (**a**) grade IV titanium (LT1), (**b**) Ti-6Al-4V alloy (LT2) and (**c**) Ti-13Nb-13Zr alloy (LT3).

The samples after laser treatment are shown in Figure 2.

Sample LT1 was characterized by the presence of two areas with different colors: blue and yellow. The LT2 sample also had local color changes. The last sample, LT3, was darker (brown) and lighter (white). The different colors of the presented samples resulted from the various thicknesses of the oxide layers formed after laser melting.

### 3.2. Microstructure of Prepared Surface 

**Figure 3:**
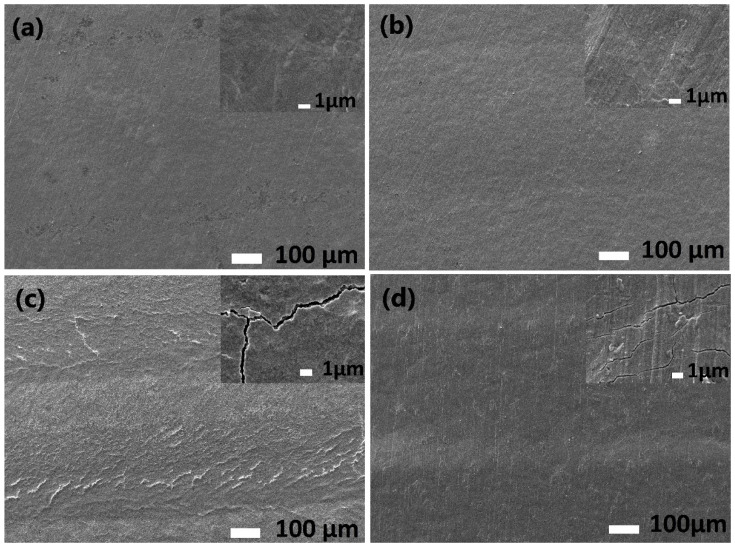
Microstructure of (**a**) LT1 sample blue area, (**b**) LT1 sample yellow area, (**c**) LT2 sample and (**d**) LT3 sample.

Figure 3 shows the scanning electron microscope photos of the samples. 

Figure 3b shows that cracks could be observed in the yellow area of the LT1 sample. In the case of the LT2 sample, the cracks form shapes similar to a honeycomb structure. In contrast, the cracks formed on the LT3 sample are parallel to each other, but of irregular length. At x10,000 magnification, cracks are observed on the LT1 surface, but its needle structure is visible. In the case of LT2, the fracture is a distinct, wider line compared to the LT3 sample. A lamellar surface structure was also noted on the latter sample.

In Figure 3c, the clear path of the laser beam was observed for LT2. On the surface of the LT1 sample (Figure 3b), a darker band can be seen in the yellow area, which is probably the laser path. In the case of the blue area on the grade IV Ti sample (Figure 3a), surface layers of the material are visible, while for the LT3 sample (Figure 3d) no clear paths of the laser beam are observed. At x1000 magnification, for the grade IV Ti surface, no cracks were observed for either of the (blue and yellow) areas. In contrast, for the LT2 and LT3 samples, cracks are visible on the surface, and for the first alloy the crack network is denser. Impurities were found on the yellow surface areas of LT1 and LT3.

### 3.3. Analysis of the Thickness of the Obtained Surface Layers

**Figure 4:**
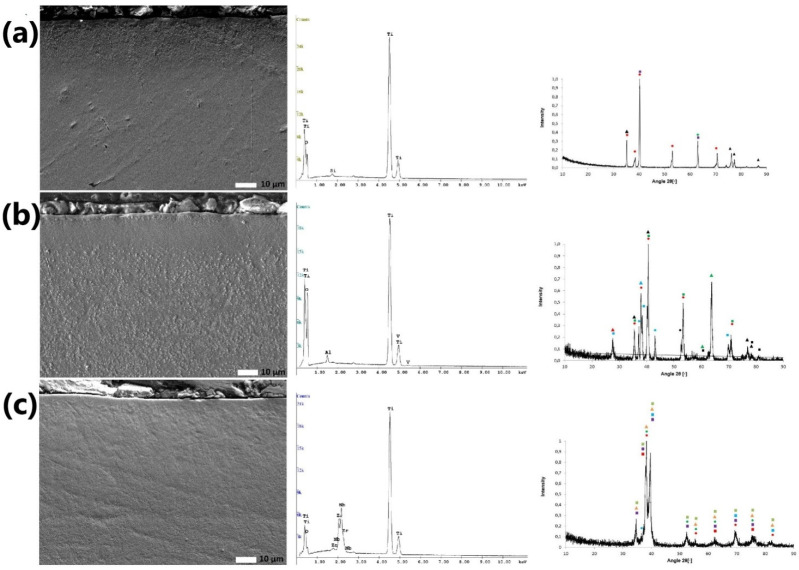
The microstructures of the cross sections of the obtained surface layers of the samples and EDS and XRD diffractograms: (**a**) LT1: ⬤—Ti, ▲—Ti6 O, ■—O0.48 Ti, ⬤—O Ti; (**b**) LT2: ■—Ti O2,▲—Ti3 O, ⬤—Ti, ■—Al Ti3, ▲—Ti6 O, ▲—Al-Ti-O2, ⬤—Ti O, ⬤—Ti2 O3, ■–V7 O3, ▲—Al8.00 V16.00 O32.00; (**c**) LT3: ⬤—Ti, ■—O0.48 Ti, ▲—O2 Zr0.958, ■—Nb6 O12 Ti12, ■—Nb, ⬤—O Ti, ■—O2 Ti.

Figure 4 shows the SEM photos of the obtained layers at a magnification of x2000 and the graphs obtained from the EDS and XRD analyses for LT1, LT2 and LT3.

In Figure 4c, an LT3 melted layer that is very thin and inhomogeneous is observed. A clear line showing the melted layer can be seen on the cross section of the LT2 sample (lighter line). Additionally, grain refinement can be observed in the middle zone. Its structure is composed of coniferous grains. In the cross section of the LT1 sample, the layer is visible as a darker band. Moreover, no cracks can be observed in the cross sections of the LT1, LT2 and LT3 samples, in contrast to the case when observing their surfaces. This may indicate that the cracks are only on the surface, i.e., only in the oxidized layer. For the LT2 sample, individual zones of the surface layer after laser treatment can be observed. Looking from the edge of the sample, the zones are subsurface, central, transition and base material.

Table 6 shows the results for the thickness of the obtained layers.

The thickest melted layer was obtained for the LT2 sample. This was 163 ± 13 µm. Due to its width, it was possible to observe all the characteristic zones. The thickness of the layer for the LT3 sample was 90 ± 6 µm and for LT1 it was 65 ± 4 µm. In these two layers, the near-surface zone was very thin.

### 3.4. Chemical Composition Analysis

After EDS testing, and based on Figure 4, it was found that the LT1 sample surface was oxidized. For LT2, an increase in oxygen content was observed in the tested surface area. The main alloy element, titanium, had the highest percentage; oxygen had about half that of titanium and the content of aluminum and vanadium had decreased. The presented research results refer only to the macroscopic description and we cannot treat them as reliable for the entire layer. Nevertheless, the decrease in the percentage of harmful elements such as aluminum and vanadium in the surface layer is evident. After testing LT3, it was found that the content of zirconium and niobium decreased in favor of the oxygen content.

### 3.5. Phase Composition Analysis

The LT1 phase diagram in Figure 4 shows that there are only phases composed of titanium and its oxides on the sample surface. This is confirmed by the tendency of titanium to oxidize in air. The passive layer of titanium oxide formed also increases the corrosion resistance [45,46].

In the case of the phase composition of the LT2 surface, the following phases were observed: Ti, Al Ti3, Ti6 O, Al-Ti-O2, V7 O3, Al8.00 V16.00 O32.00. The most common phases were: Ti, Al Ti3 and Ti6 O. The diffractogram confirms the formation of intermetallic phases in the Ti-Al system and the presence of vanadium oxide [5].

The phase diagram of the LT3 surface shows the following phases: O0.48 Ti, O2 Zr0.958, Nb6 O12 Ti2, O2 Ti, Nb, Ti and O Ti. The obtained diffractogram confirms the formation of titanium oxide after laser modification [7]. It is also shown that zirconium or niobium titanate oxides are formed.

### 3.6. Roughness of Obtained Layer

Table 7 shows the roughness results (averaged from three measurements) for samples of titanium and its alloys. Their standard deviations were also determined.

After conducting the roughness test, it was found that the surface of the titanium sample (LT1) had the lowest value of the parameter Ra. It was found to have the lowest roughness compared to the other samples. The values of the Ra parameter for the LT2 and LT3 samples are comparable, at 0.60 ± 0.03 [µm] and 0.59 ± 0.01 [µm], respectively. 

### 3.7. Contact Angle Analysis

Table 8 shows the average values of the contact angle for samples BM1–BM3 and LT1–LT3. 

The lowest value of the average contact angle was obtained for LT2, at 64.63 ± 0.22°. The highest value of the contact angle was achieved for the LT1 sample, at 95.35 ± 3.66°. 

### 3.8. Nanoindentation Tests

**Figure 5:**
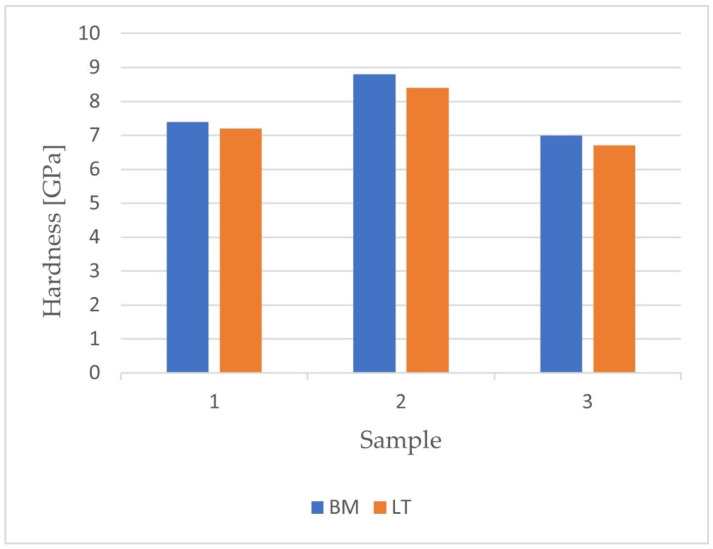
The results for hardness obtained after nanoindentation tests.

**Figure 6:**
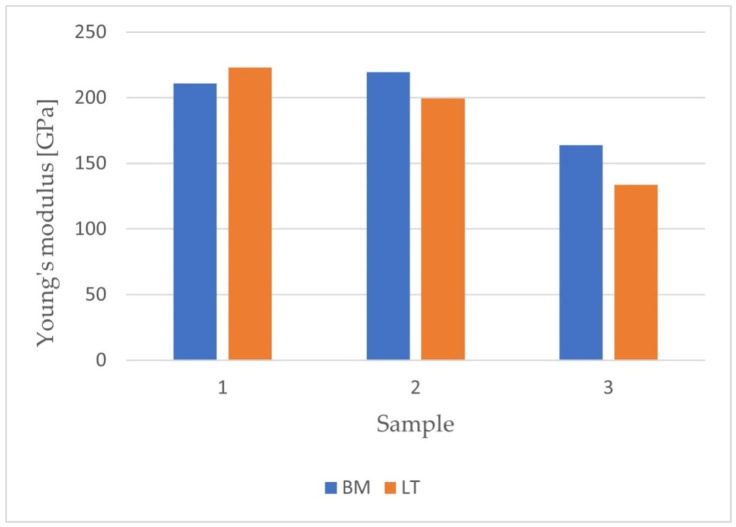
The results for Young’s modulus obtained after nanoindentation tests.

After carrying out the nanoindentation tests, the mean values of the Young’s modulus and the hardness of the remelted layers were calculated, together with their uncertainties, from the formula for standard deviation. The results are presented in Figure 5 and Figure 6.

It was found that the laser treatment increased the value of the elastic modulus of the surface layer only for the grade IV Ti sample (before: (211.0 ± 32.7) GPa, after: (223.2 ± 68.3) GPa). Such a modification causes an increase in surface differentiation, as evidenced by higher measurement uncertainty. This is particularly illustrated for the titanium sample, where the measured uncertainty of the Young’s modulus of the surface layer after laser treatment was twice as high as before. In the case of layers obtained for the samples from the titanium alloys Ti-6Al-4V and Ti-13Nb-13Zr, the laser treatment resulted in a decrease in the value of the elastic modulus. The modification of the surface makes it possible to approximate the value of the Young’s modulus for bone. Additionally, in both layers, similarly to the layer obtained for the grade IV Ti sample, an increase in the measurement uncertainty was noted.

The value of the hardness of the layer after laser treatment decreased (6.7 ± 1.6 GPa) compared to the hardness before modification (7.0 ± 1.4 GPa).

For samples made of titanium and Ti-6Al-4V alloy, the hardness of the obtained layers before remelting was 8.8 ± 1.6 GPa and after remelting was 8.4 ± 2.9 GPa. The nanoindentation tests showed that a decrease in the hardness value was noted after laser modification. It is possible that the force applied during the nanoindentation process was relatively high. As a result, the near-surface zone broke, and the indenter was pressed down to the middle zone of the remelted layer. Hence, the obtained values of hardness for the grade IV Ti and Ti-6Al-4V samples after laser modification were lower than the values before treatment. 

In the case of samples made of titanium and Ti-6Al-4V, it was found that the laser modification resulted in a decrease in the maximum deformation with the maximum load force unchanged. In contrast, for the Ti-13Nb-13Zr sample, no significant influence of the laser treatment on the relationship between the load and the deformation was observed.

### 3.9. Assessment of the Stress State of Samples after Laser Treatment

To determine the state of stress in the surface layer of laser-treated samples, a nanoindentation test was carried out before and after heat treatment. In both cases, two series of measurements were performed for each of the samples, using loads of 50 mN and 200 mN, to determine the effect of the applied force on the values of the stresses obtained. 

**Figure 7:**
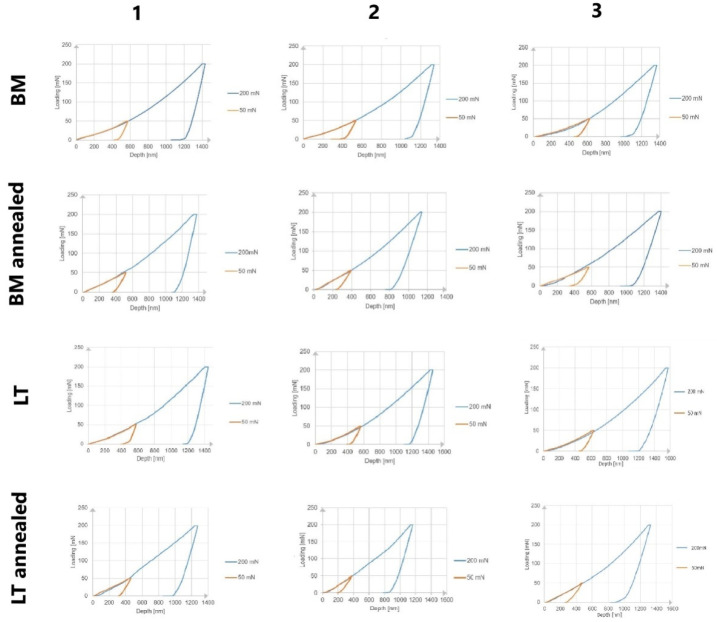
Indentation curves of samples BM and LT loaded with a maximum force of 50 and 200 mN.

Figure 7 shows the indentation curves of individual samples for both loading forces.

For each of the materials, a load–strain hysteresis curve was plotted during the indentation measurement. The shape of the unloading curve directly influences the mechanical properties of the material, as determined by the Oliver–Pharr method. The unloading curve starts at the value of the maximum depth for which the indenter is in contact with the sample surface. 

The graphs show a change in the slope of the curve before and after the heat treatment. The change in the load–depth characteristics indicates a change in the stress state. For the melted samples, greater deformation was noted under loading with the maximum force than in the case of annealed samples. This relationship occurred for all tested materials.

On the basis of the obtained results, the mean values of the forces causing the same cavity were determined, in order to determine the stresses at the same depth. Table 9 lists the average loading force, the measurement depth and the area of the instantaneous indenter impression for each material.

The calculations of residual stresses were performed in accordance with the presented methodology. The changes related to the stress state that occured in the surface layer after heat treatment are presented in Table 10.

A greater reduction in the residual stresses after stress relief can be observed for measurements made at a depth of 300 nm. Hence, it is concluded that the stresses decrease deeper into the surface layer. The greatest change in the value of residual stresses was recorded for the LT2 samples and the smallest for the LT1 samples. There were no visible differences in the stress values between the measurements with maximum forces of 50 and 200 mN for LT1 and LT2 at a depth of 300 nm. However, the difference in the case of LT3 was as high as 1.3 GPa. The negative values of residual stresses prove that the stresses occurring before the heat treatment were tensile. 

**Figure 8:**
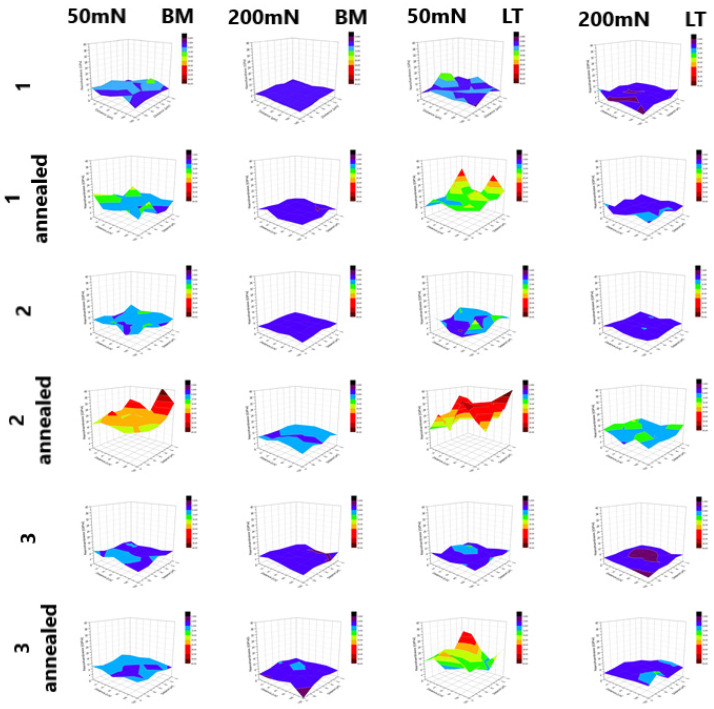
Three-dimensional hardness maps of the BM, LT and annealed BM and LT samples.

The stresses occurring in the material affect its hardness. Figure 8 presents maps of the hardness distributions of reference samples BM, remelted LT samples and annealed samples. 

**Figure 9:**
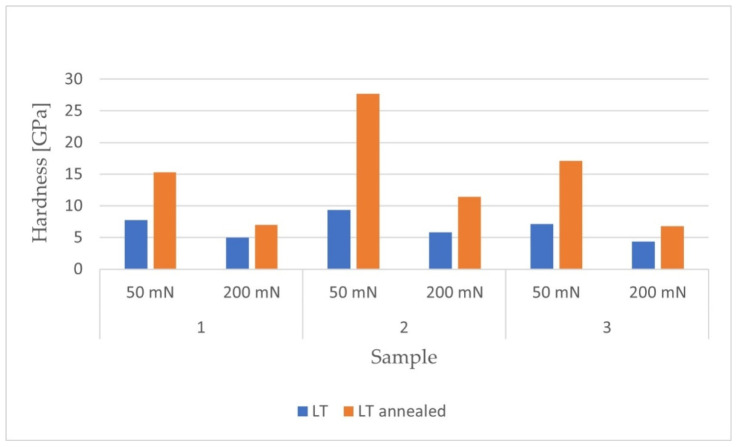
Values of hardness obtained after melting for two maximum loadings: 50mN and 200 mN.

Figure 9 shows the numerical values of the hardness obtained after melting.

For each of the materials, an increase in hardness was observed with a decrease in penetration depth. The remelted Ti-6Al-4V (LT2) sample had the highest hardness values. The hardness distributions of the materials loaded with a maximum force of 50 mN showed greater unevenness in the obtained results compared to samples loaded with a maximum force of 200 mN. The internal stresses generated in the surface layer were tensile. The reduction of these stresses as a result of the annealing process led to an increase in the hardness of all tested materials.

### 3.10. Assessment of the Stress state of Reference Samples

In order to perform a detailed analysis of the influence of laser modification on the state of residual stress, the obtained results were compared to the annealed samples of the base material. The load–strain hysteresis plot for the reference samples is shown in Figure 7.

As for the materials subjected to laser modification, changes in the slope of the curves and the value of the maximum cavity after heat treatment were observed. However, they were less pronounced, especially for the Ti-13Nb-13Zr alloy and titanium, indicating a much lower reduction of stresses in the annealed non-melted materials.

Table 11 summarizes the data for the stress state analysis. These are the adopted depths, the surface areas of the Berkovich imprint and the average force values calculated on the basis of all measurements.

The changes related to the state of the stresses that occurred in the surface layers of the base materials after heat treatment are summarized in Table 12.

The presented calculation results for the maximum load of 200 mN confirm that the residual stresses decrease deeper into the surface layer. In the case of the reference materials, a greater influence of the maximum force on the obtained stress values was noted than for the melted samples. As in the case of the laser-treated materials, the highest stress value was observed for the Ti-6Al-4V alloy.

As in the case of the laser-remelted samples, the Ti-13Nb-13Zr alloy was characterized by the highest hardness. The presented 3D distributions for each of the samples confirmed the dependence of the increase in hardness on the decrease in the depth of penetration into the surface. The obtained hardness distributions for the samples tested with a maximum force of 50 mN were also more uneven in the case of the reference materials. For each of the tested materials, higher hardness values were observed for the laser-modified samples. The residual stresses in the samples before heat treatment were tensile. After stress relief annealing, an increase in hardness was obtained in all tested materials.

**Figure 10:**
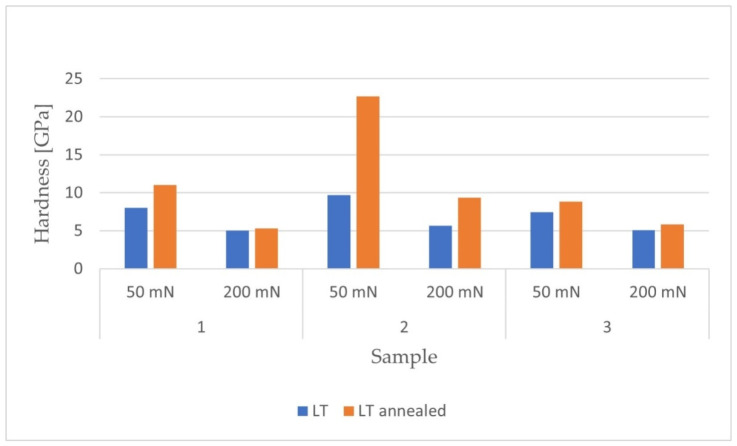
Results for hardness measurements for two maximum loadings: 50mN and 200 mN.

The numerical values for the obtained hardness of the reference materials are summarized in Figure 10.

As in the case of the laser-remelted samples, the Ti-6Al-4V sample was characterized by the highest hardness. The hardness distributions for the samples tested with a maximum force of 50 mN were also more uneven for the reference materials. For each of the samples, higher hardness was observed for the laser-modified samples. The residual stresses in the samples before heat treatment were tensile. After stress relief annealing, an increase in hardness was obtained in all tested materials.

### 3.11. Influence of Laser and Heat Treatment on Residual Stress

**Figure 11:**
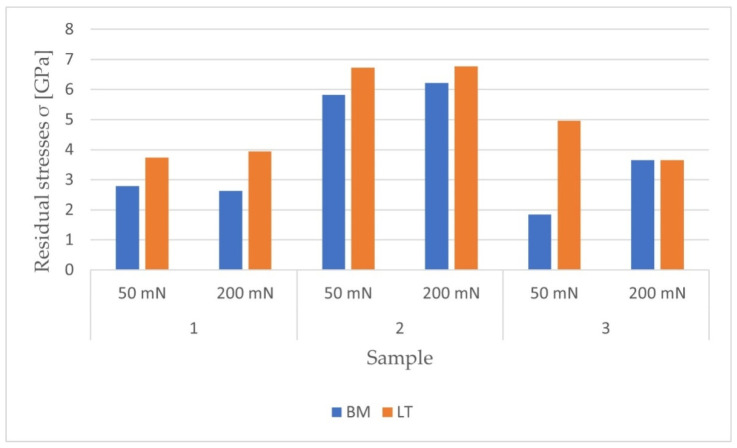
Residual stresses obtained at a depth of 300 nm from the surface of the materials.

**Figure 12:**
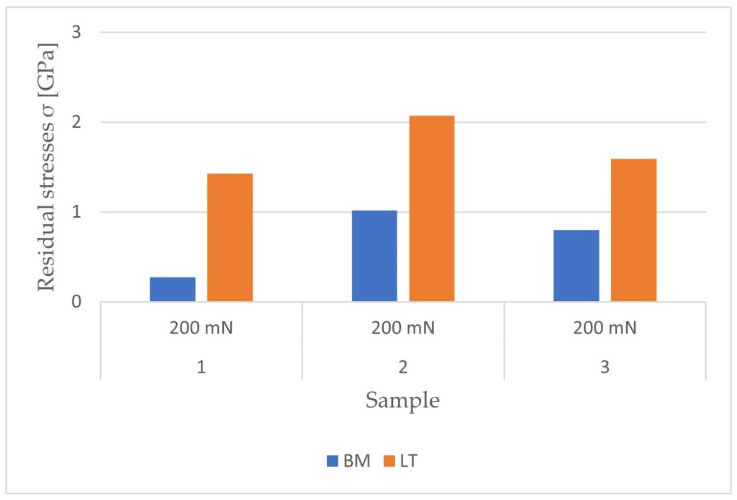
Residual stresses obtained at a depth of 1000 nm from the surface of the materials.

Figure 11 and Figure 12 show the obtained stresses and their character determined at depths of 300 and 1000 nm.

The obtained results indicate an increase in residual tensile stress after remelting. By averaging the results obtained from all measurements, the effect of treatment for individual materials was determined. Modification of the pure titanium increased the stresses by 225%, with the most intense stresses in the deeper sections of the surface layer. For the Ti-6Al-4V alloy, the stress increase was recorded as 59.4%; however, this material had the highest initial internal stresses. In the case of the Ti-13Nb-13Zr alloy, a greater stress increase occurred at a depth of 300 nm when a maximum load of 50 mN was applied. The overall increase in stress after remelting this sample was 133.5%.

Based on the results presented in Figure 11, the influence of the maximum force value on the obtained stresses was analyzed in the nanoindentation test. For pure titanium and Ti-6Al-4V, there were no significant differences in the stress states, unlike Ti-13Nb-13Zr. For measurements with a maximum force of 200 mN at a depth of 300 nm, the average stress value was 3.65 GPa both before and after laser processing. Thus, no change in the stress state was noted. However, for the measurement with a Pmax of 50 mN, the difference was over 3.1 GPa. Measuring the same sample at a depth of 1000 nm using a maximum force of 200 mN showed a similar increase in stress as that measured with a force of 50 mN at a 300 nm indentation. Although in the remaining cases the results do not differ significantly, more reliable results for the residual stresses at smaller depths are obtained by using a lower maximum force. 

**Figure 13:**
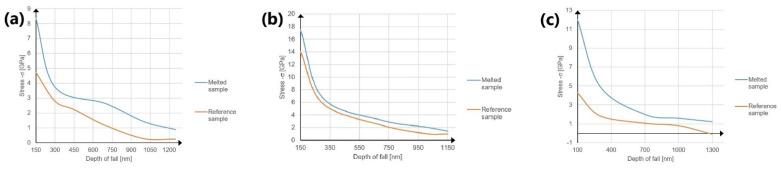
Depth of fall in (**a**) pure titanium (grade IV Ti), (**b**) Ti-6Al-4V alloy and (**c**) Ti-13Nb-13Zr alloy.

On the basis of averaged results of measurements made with a maximum load of 200 nm, the residual stresses were calculated at different depths from the surface at intervals of 200 nm. The dependence of the stress values on the depth in the material for individual samples tested with a maximum force of 200 mN are shown in Figure 13.

The obtained diagrams confirm that the stresses decrease with depth into the surface layer. For the non-laser-modified Ti-13Nb-13Zr sample at a depth of 1300 nm, a change in the nature of the residual stress from a tensile stress to a compressive stress of 99 MPa was observed. However, after laser processing only tensile stresses were obtained, as in the other materials.

## 4. Discussion

The conducted research allowed for a detailed analysis of the laser-remelted layer of pure titanium and its alloys (Ti-6Al-4V and Ti-13Nb-13Zr). During the macroscopic evaluation, areas of different colors could be observed on the surfaces of the samples. Chemical composition analysis (EDS) showed that titanium oxides were formed on the surface, and the percentage of oxygen influenced the colors of the obtained molten layers. Blue-colored titanium sample layers indicate the formation of anatase (a type of titanium oxide), while yellow-colored areas may suggest the formation of a different type of titanium oxide, i.e., brookite, which is less durable. In the case of the Ti-13Nb-13Zr sample, the obtained brown area is probably rutile which has been contaminated with niobium (hence the color).

The Ti-6Al-4V and Ti-13Nb-13Zr samples had a more cracked surface than the pure titanium sample. The formed cracks were superficial and were not visible in the cross sections. However, if these alloys are to be used for implants, research to verify the brittleness of the subsurface zones should be extended. Crumbles resulting from microcracks may adversely affect the body (e.g., inducing immune reactions) [47]. After laser treatment, titanium and titanum alloys in the near-surface zone of an amorphous structure were observed; in the middle zone of the surface layer a fragmented acicular martensitic structure was formed.

The highest thickness of the melted layer was obtained for the Ti-6Al-4V sample, making it possible to determine its individual zones. The melted layers of the remaining samples were thinner and a microhardness distribution analysis could not be performed for these. The near-surface zone of the Ti-13Nb-13Zr and titanium layers were so thin that they cracked when tested for microhardness. 

Laser treatment had a positive effect on the surface of the Ti-6Al-4V sample, as it reduced the percentage of harmful elements (aluminum and vanadium). Therefore, it is possible to reduce the side effects associated with the use of this alloy in implantology. However, since the examination of chemical composition is a qualitative analysis, it is necessary to study this aspect further, e.g., in biological research.

Examination of the contact angle of the grade IV Ti sample surface showed that it was greater than 90°. This means that the obtained surface layer was hydrophobic, i.e., only slightly wetting. The surfaces of the samples made of Ti-6Al-4V and Ti-13Nb-13Zr obtained an average wetting angle below 90°, and so were hydrophilic and showed good wetting properties. In the use of titanium and its alloys for implants, the aim is to obtain a hydrophilic or even super-hydrophilic surface. This is because the highest bioactivity and bioadhesion after implant application occur with a contact angle in the range of 0° to 90°. This is particularly important for the success of the osseointegration process, as osteoblasts and other osteogenic cells adhere more easily to a highly wettable surface [48,49]. It was found that the best material among the tested samples was Ti-6Al-4V, because it had the smallest contact angle of 64.63°. Accordingly, a sample made of pure titanium does not meet the surface requirements for implant applications. 

The analyses of the phase composition confirmed the formation of titanium oxides in the remelted layer [7], which increase the corrosion resistance. The Ti-6Al-4V and Ti-13Nb-13Zr sample diffractograms also indicated the formation of vanadium oxide and zirconium oxide, respectively. The analysis of the results also showed that one of the most common phases in the Ti-6Al-4V layer was the intermetallic phase from the Ti-Al system.

The most favorable roughness parameters were obtained for the Ti-6Al-4V sample. The optimal value of the Ra parameter for implants is 1–2 µm [13] or 1–10 µm [10]. Due to the roughness, the osseointegration process is faster. The obtained results did not fall within the given range. The highest value of the Ra parameter was obtained for the Ti-6Al-4V sample at Ra = 0.60. A similar value of Ra = 0.59 was obtained for the sample made of Ti-13Nb-13Zr. It is believed that changing the laser processing parameters (e.g., applying more power) could increase the roughness parameter value to within the desired range for implant applications. Higher surface roughness enables a more efficient osseointegration process and increases the enzymatic activity of osteoblasts [50,51]. The highest value of this parameter was obtained for the Ti-6Al-4V sample surface, and at the same time, this was closest to the expected value. It should also be remembered that a too-high Ra parameter may affect, among other things, the release of ions between bone and titanium.

The laser modification had a beneficial effect on the modulus of elasticity of the layers of the remelted titanium alloys: Ti-6Al-4V (199.6 ÷ 47.9 GPa) and Ti-13Nb-13Zr (133.7 ÷ 27.8 GPa). A silmilar relationship was observed for Ti-13Nb-13Zr in [12]. Their values decreased, which means that the titanium alloys studied could be used in implantology. The aim is to obtain biomaterials with a low Young’s modulus, albeit still distant from the value of the modulus of elasticity of human bone (25.8 GPa) [52]. Efforts should be made to modify the parameters used during machining to obtain the desired results.

The tests showed that the laser treatment performed affected the stress state of the tested materials. The nature of the residual stresses achieved may be due to many factors, inter alia:

- the processing parameters used, especially the laser heat input;
- the degree of oxidation of the samples, which may be related to insufficient shielding gas flow or an incorrect nozzle setting;
- the limited heat capacity of the samples resulting from their small size (subsequent laser passes were made on a highly heated surface);
- significant internal stresses after cutting and grinding samples, superimposed upon the thermo-structural stresses arising during laser processing.

High tensile stresses in the near-surface layer reduce the operational properties. 

They increase the tendency towards, and the risk of, microcracking on the surface of materials, which may in turn lead to a reduction in fatigue strength, corrosion resistance and tribological properties [47,53]. The microcracks accompanying the cracks can also trigger an immune response in the body.

The resulting tensile stresses were effectively removed by the applied heat treatment. The analysis of studies on similar subjects shows that as a result of Nd: YAG laser treatment using the laser shock peening (LSP) method, high tensile stresses are observed in the subsurface zone of the surface layer [47]. In [54] a positive effect of LSP modification on the fatigue cracking delay of Ti-5Al-2Sn-4Mo-2Zr-6Mo alloy was observed. The mechanism of crack initiation inhibition is described, based on the change in the plastic zone size and the decrease in the crack propagation energy density at its tip. The beneficial effect of the compressive residual stresses on the reduction of the size of the plastic zone was also demonstrated by reducing the size of the applied load, which is responsible for fatigue crack propagation.

Well-chosen annealing parameters allow stresses to be reduced, maintaining the obtained material structure. Stress relief certainly reduces the risk of microcracking and increases the service life of the manufactured layers.

The stress relief annealing process was correct, as evidenced by the change in the stress state of the tested materials. Taking into account the thickness of the sample cross sections, the holding time for pure titanium and Ti-6Al-4V can be shortened to one hour, as in the case of Ti-13Nb-13Zr. This change should not affect the efficiency of the heat treatment, and it reduces the overall processing time. In addition, in [55] the author suggests cooling α + β alloys with a small share of the β-phase (which includes the Ti-6Al-4V alloy) in air, because slow cooling in a furnace may favor the formation of Ti3Al, which reduces the alloy’s resistance to stress corrosion.

Taking into account all the above-mentioned conclusions, it was found that the laser treatment with the applied parameters had the most favorable effect on the surface of the Ti-6Al-4V sample. After subsequent biological and cellular research, it may be possible to use this to modify the surface of implant materials.

A perspective on the direction of positive changes in this regard may be an increase in the heat input value (an increase in the laser power and a reduction in the speed of the process), which may result in obtaining the desired surface roughness for implantology applications and reducing the tensile stress value. 

## 5. Summary

Among the three tested Ti materials, medically pure titanium and Ti-6Al-4V and Ti-13Nb-13Zr alloys, the Ti-Al-V alloy demonstrated the best properties after laser treatment. In particular, this alloy showed highest melted layer thickness, the highest hardness and the most advantageous roughness parameters.

The laser treatment had different effects on the microstructure and the properties of the surface layers for different process parameters, such as the remelting parameters, the degree of oxidation of the samples, the limited heat capacity of the samples and significant internal stresses after cutting and grinding samples, which may be attributed to the complex heat transfer paths in the surface layer resulting in phase transformations and the appearance of thermal stresses.

Annealing had positive effect on the behavior of the layers as it lowered the levels of intrinsic stress and increased the hardness in all test materials, thus increasing the service life.

## Figures and Tables

**Table 1:** Selected mechanical properties of grade IV Ti, Ti-6Al-4V and Ti-13Nb-13Zr samples (based on the manufacturers’ certificates).

Material	Sample	Microstructure	Delivery Condition	A [%]	R_0.2_ [MPa]	R_m_ [MPa]
Grade IV Ti	BM1	α	annealed	15	480	550
Ti-6Al-4V	BM2	α + β	annealed	14	880	950
Ti-13Nb-13Zr	BM3	β	annealed	15	710	840

**Table 2:** The chemical composition of samples (based on the manufacturers’ certificates).

Sample	Al	V	Zr	Nb	Fe	C	O	N	H	Ti
BM1	-	-	-	-	0.50	0.08	0.4	0.05	0.015	rest
BM2	5.50–6.75	3.50–4.50	-	-	0.30	0.10	0.2	0.05	0.0125	rest
BM3	-	-	13.49	13.18	0.085	0.035	0.078	0.007	0.004	rest

**Table 3:** The parameters of laser treatment.

Average Power of the Laser Beam	Pulse Power	Time	Speed of Laser Beam	Frequency	Overlapping
25 W	100 W	1 ms	60 mm	25 Hz	50%

**Table 4:** The parameters of nanoindentation tests.

Parameter	Value
Number of measurements	25 (5 × 5)
Maximum force [mN]	50
Loading time [s]	20
The dwell period at maximum load [s]	5
Unloading time [s]	20
Retraction distance [µm]	30

**Table 5:** Stress relief parameters.

Sample	Material	Annealing Temperature [°C]	Annealing Time [h]	Heating Time [h]	Cooling Time [h]
LT1	Grade IV Ti	600	6	6	9
LT2	Ti-6Al-4V	600	6	6	9
LT3	Ti-13Nb-13Zr	500	1	5	9

**Table 6:** Thickness of the obtained surface layers for individual samples.

Sample	Thickness [µm]
LT1	65 ± 4
LT2	163 ± 13
LT3	90 ± 6

**Table 7:** Results for properties of roughness.

Parameter [µm]	Sample	BM	LT
Ra	1	0.12 ± 0.01	0.38 ± 0.02
	2	0.02 ± 0.01	0.60 ± 0.03
	3	0.02 ± 0.01	0.59 ± 0.01

**Table 8:** The contact angle values.

	Contact Angle [°]	
Sample	BM	LT
1	55.79 ± 0.80	95.35 ± 3.66
2	72.87 ± 1.10	64.63 ± 0.22
3	46.20 ± 0.90	87.35 ± 2.25

**Table 9:** Values adopted for the calculation of residual stresses.

Material	Maximum Loading [mN]	Depth of Measurement [nm]	Average Force at the Assumed Depth [mN]	Indenter Imprint Area [nm^2^]
LT1	50	300	21.908	2.2041
	200	300	20.157	2.2041
		1000	115.218	24.490
LT2	50	300	22.332	2.2041
	200	300	21.464	2.2041
		1000	128.414	24.490
LT3	50	300	19.123	2.2041
	200	300	17.194	2.2041
		1000	99.965	24.490
LT1 annealed	50	300	30.156	2.2041
	200	300	28.843	2.2041
		1000	150.133	24.490
LT2 annealed	50	300	37.165	2.2041
	200	300	36.382	2.2041
		1000	179.154	24.490
LT3 annealed	50	300	30.060	2.2041
	200	300	25.245	2.2041
		1000	138.890	24.490

**Table 10:** List of calculated values of residual stresses of remelted samples.

Material	Maximum Loading [mN]	Depth of Measurement [nm]	Stress Value [GPa]
LT1	50	300	−3.742
	200	300	−3.940
		1000	−1.426
LT2	50	300	−6.734
	200	300	−6.768
		1000	−2.072
LT3	50	300	−4.962
	200	300	−3.653
		1000	−1.589

**Table 11:** Values adopted for the calculation of residual stresses.

Material	Maximum Loading [mN]	Depth of Measurement [nm]	Average Force at the Assumed Depth [mN]	Indenter Imprint Area [nm^2^]
BM1	50	300	21.914	2.2041
	200	300	21.196	2.2041
		1000	120.615	24.490
BM2	50	300	24.596	2.2041
	200	300	21.935	2.2041
		1000	126.620	24.490
BM3	50	300	19.947	2.2041
	200	300	18.228	2.2041
		1000	115.611	24.490
BM1 annealed	50	300	28.063	2.2041
	200	300	26.988	2.2041
		1000	127.392	24.490
BM2 annealed	50	300	37.436	2.2041
	200	300	35.642	2.2041
		1000	151.623	24.490
BM3 annealed	50	300	24.023	2.2041
	200	300	26.273	2.2041
		1000	135.151	24.490

**Table 12:** List of calculated values of residual stresses of annealed samples.

Material	Maximum Loading [mN]	Depth of Measurement [nm]	Stress Value [GPa]
BM1 annealed	50	300	−2.789
	200	300	−2.627
		1000	−0.276
BM2 annealed	50	300	−5.825
	200	300	−6.219
		1000	−1.020
BM3 annealed	50	300	−1.849
	200	300	−3.650
		1000	−0.798

## Data Availability

Not applicable.
